# Docetaxel-induced prostate cancer cell death involves concomitant activation of caspase and lysosomal pathways and is attenuated by LEDGF/p75

**DOI:** 10.1186/1476-4598-8-68

**Published:** 2009-08-28

**Authors:** Melanie Mediavilla-Varela, Fabio J Pacheco, Frankis Almaguel, Jossymar Perez, Eva Sahakian, Tracy R Daniels, Lai Sum Leoh, Amelia Padilla, Nathan R Wall, Michael B Lilly, Marino De Leon, Carlos A Casiano

**Affiliations:** 1 Center for Health Disparities and Molecular Medicine, Loma Linda University School of Medicine, Loma Linda, CA 92350, USA; 2 Department of Basic Sciences, Loma Linda University School of Medicine, Loma Linda, CA 92350, USA; 3 Department of Biological Sciences, Centro Universitário Adventista de São Paulo, São Paulo, Brazil; 4 Division of Surgical Oncology, Department of Surgery, David Geffen School of Medicine, University of California, Los Angeles, CA 90095, USA; 5 Chao Family Comprehensive Cancer Center, University of California, Irvine, CA 92868, USA; 6 Department of Medicine, Loma Linda University School of Medicine, Loma Linda, CA 92350, USA

## Abstract

**Background:**

Hormone-refractory prostate cancer (HRPC) is characterized by poor response to chemotherapy and high mortality, particularly among African American men when compared to other racial/ethnic groups. It is generally accepted that docetaxel, the standard of care for chemotherapy of HRPC, primarily exerts tumor cell death by inducing mitotic catastrophe and caspase-dependent apoptosis following inhibition of microtubule depolymerization. However, there is a gap in our knowledge of mechanistic events underlying docetaxel-induced caspase-independent cell death, and the genes that antagonize this process. This knowledge is important for circumventing HRPC chemoresistance and reducing disparities in prostate cancer mortality.

**Results:**

We investigated mechanistic events associated with docetaxel-induced death in HRPC cell lines using various approaches that distinguish caspase-dependent from caspase-independent cell death. Docetaxel induced both mitotic catastrophe and caspase-dependent apoptosis at various concentrations. However, caspase activity was not essential for docetaxel-induced cytotoxicity since cell death associated with lysosomal membrane permeabilization still occurred in the presence of caspase inhibitors. Partial inhibition of docetaxel-induced cytotoxicity was observed after inhibition of cathepsin B, but not inhibition of cathepsins D and L, suggesting that docetaxel induces caspase-independent, lysosomal cell death. Simultaneous inhibition of caspases and cathepsin B dramatically reduced docetaxel-induced cell death. Ectopic expression of lens epithelium-derived growth factor p75 (LEDGF/p75), a stress survival autoantigen and transcription co-activator, attenuated docetaxel-induced lysosomal destabilization and cell death. Interestingly, LEDGF/p75 overexpression did not protect cells against DTX-induced mitotic catastrophe, and against apoptosis induced by tumor necrosis factor related apoptosis inducing ligand (TRAIL), suggesting selectivity in its pro-survival activity.

**Conclusion:**

These results underscore the ability of docetaxel to induce concomitantly caspase-dependent and independent death pathways in prostate cancer cells. The results also point to LEDGF/p75 as a potential contributor to cellular resistance to docetaxel-induced lysosomal destabilization and cell death, and an attractive candidate for molecular targeting in HRPC.

## Introduction

Prostate cancer (PCa) is the most frequently diagnosed cancer in men and the second leading cause of male cancer deaths in the U.S. [1]. PCa also presents the greatest racial disparity of any cancer in the U.S., with higher incidence and mortality in African-American men (AA), compared to other ethnic groups [2,3]. A factor contributing to these disparities is the more aggressive and perhaps more therapy-resistant form of the disease observed among AA men [2,3]. Understanding the underlying causes of this increased tumor aggressiveness would require a multi-prong approach that includes evaluation of potential racial/ethnic differences in prostate tumor biology, identification of gene-environment interactions leading to prostate inflammation, elucidation of molecular mechanisms associated with PCa chemoresistance, and development of more effective therapeutic interventions for HRPC.

Docetaxel (DTX, Taxotere^®^), a semi-synthetic analog of paclitaxel, has emerged in recent years as the standard of care for chemotherapy of HRPC [4]. Unfortunately, most HRPC patients treated with DTX ultimately manifest resistance to the drug and succumb to the disease. The mechanisms underlying resistance to DTX in HRPC appear to be diverse and poorly understood; however, a growing body of evidence implicates cellular anti-apoptotic, stress, and redox signaling pathways in the development of HRPC and DTX resistance [5-10]. Attaining a mechanistic understanding of DTX-induced cell death and DTX resistance in PCa would facilitate the identification of new molecular targets and the development of rational therapeutic strategies aimed at sensitizing HRPC to this and other anti-tumor drugs.

It is generally accepted that DTX primarily exerts tumor cell death by inducing mitotic catastrophe and caspase-2 and -3-dependent apoptosis following inhibition of microtubule depolymerization [11-16]. DTX has also been reported to induce non-apoptotic death in tumor cells, both in vitro and in vivo, depending on the dose, cell type, and tumor microenvironment [11,15,17]. While mechanistic insights into non-apoptotic, caspase-independent cell death induced by paclitaxel have been reported [18,19], knowledge of mechanistic events underlying DTX-induced caspase-independent cell death is very scarce. Caspase-dependent and independent cell death pathways co-exist in tumor cells and can be triggered in parallel by therapeutic agents [20-22]. While most efforts in targeting cellular survival pathways have focused on inactivating proteins that antagonize caspase-dependent pathways, there is growing consensus that targeting survival proteins that antagonize caspase-independent or non-apoptotic cell death might be a promising strategy for increasing the effectiveness of chemotherapeutic drugs [20-22].

The lens epithelium derived growth factor p75 (LEDGF/p75) is emerging as a stress response protein that promotes cell survival against death induced by stressors such as oxidative stress, heat shock, serum starvation, and chemotherapy [23-28]. This protein is also known as transcription co-activator p75 (TCP75), PC4 and SFRS1 interacting protein (PSIP), and dense fine speckled autoantigen of 70 kD (DFS70) [29-31]. LEDGF/p75 has been identified as a member of the hepatoma-derived growth factor family [32], a NUP98-fusion protein resulting from chromosomal translocations in leukemias [33], an autoantigen in diverse autoimmune and inflammatory conditions [30,34-36], and a key cellular co-factor for the chromosomal integration of HIV-1 [37-39]. LEDGF/p75 is presumed to promote malignant transformation and resistance to stress-induced cell death via either direct binding to promoter regions of stress survival and anti-oxidant genes, or protein-protein interactions leading to transcriptional activation of cancer-related genes [40-42]. The stress-survival activity of LEDGF/p75 appears to be regulated by alternative splicing resulting in the removal of its carboxyl C-terminal domain, and by caspase-mediated disruption of both its amino (N) and C-terminal domains [25,43].

We reported previously that LEDGF/p75 is the target of autoantibody responses in some patients with PCa, and that its expression is upregulated in advanced stage PCa [44]. LEDGF/p75 expression was also found elevated in human breast and bladder carcinomas, and its ectopic overexpression increased the tumorigenic potential of human cancer cells in murine models [27]. In this study we provide evidence that treatment of HRPC cells with DTX, in addition to inducing mitotic catastrophe and caspase-dependent apoptosis, induces a caspase-independent cell death pathway that involves lysosomal destabilization and cathepsin B activation. We also show that ectopic overexpression of LEDGF/p75 attenuates DTX-induced lysosomal destabilization and cell death, but not DTX-induced mitotic catastrophe or apoptosis induced by tumor necrosis factor related apoptosis inducing ligand (TRAIL). These results underscore the ability of DTX to activate multiple cell death pathways, and point to LEDGF/p75 as a potential contributor to DTX resistance in PCa.

## Materials and methods

### Cell Lines, Antibodies, and Reagents

PC3, DU145 and RWPE-2 cell lines were obtained from American Type Culture Collection (ATCC) and cultured according to ATCC recommendations. The following antibodies were used: mouse monoclonal anti-poly (ADP-ribose) polymerase (PARP) AB-2 (Calbiochem), mouse monoclonal anti-β-actin (Sigma-Aldrich), goat polyclonal anti-cathepsin B (Santa Cruz Biotechnology), and horseradish peroxidase (HRP)-labeled secondary IgG antibodies (Zymed). Human autoantibodies to LEDGF/p75 were a kind gift from Dr. Edward Chan (University of Florida, Gainesville). The broad caspase inhibitor benzylocarbonyl-Val-Ala-Asp-fluoromethyl ketone (Z-VAD-FMK) and the specific caspase-2 inhibitor N-acetyl-Val-Asp-Val-Ala-Asp-aldehyde (Ac-VDVAD-CHO) were purchased from Biomol International. DTX was purchased from LC Laboratories. Recombinant human TRAIL and actinomycin D were purchased from R&D Systems. Staurosporine (STS), N-acetyl-Asp-Glu-Val-Asp-7-amino-4-methylcoumarin (Ac-DEVD-AMC, fluorogenic caspase-3/7 substrate), and Ac-VDVAD-AMC (fluorogenic caspase-2 substrate) were purchased from Axxora. Inhibitors of cathepsin B (CA-074Me), cathepsin L (cathepsin L inhibitor I) and cathepsin D (pepstatin A) were obtained from EMD Biosciences. The cathepsin B fluorogenic substrate Magic Red MR-(RR)_2_, Acridine Orange (AO), and Hoescht 33342 were purchased from Immunochemistry.

### Induction of Cell Death

Cell death was induced by treatment with DTX (various concentrations), TRAIL/actinomycin D (100 ng/ml each), or 4 μM STS for up to 48 h. In some experiments cells were preincubated with either 100 μM of Z-VAD-FMK, 100 μM of Ac-VDVAD-CHO, 100 μM cathepsin B inhibitor, 150 μM cathepsin L inhibitor, or 100 μM cathepsin D inhibitor for 1 h prior to exposure to the cytotoxic drugs. Cells were visualized on an Olympus IX70 microscope equipped with Hoffmann Modulation Contrast (Olympus American). Images were acquired using a digital Spot Imaging System (Diagnostic Instruments).

### Cell Viability Assays

Cells seeded in 96-well plates (10^4 ^cells per well) were treated with cytotoxic drugs in the presence and absence of inhibitors, washed with phosphate buffered saline (PBS), and fixed in 4% paraformaldehyde for 1 h at 4°C. Cells were then washed three times with distilled water, and Accustain Crystal Violet solution (Sigma-Aldrich) (1:4) was added to each well followed by incubation for 20 minutes at room temperature. Plates were washed with distilled water to remove excess dye and then dried at room temperature. Acetic acid (10% v/v) was added to each well for 10 minutes and absorbance was measured at 570 nanometers (nm) using a μQuant microplate reader (Bio-tek Instruments). Cell viability was also determined using a modified (3-(4,5-dimethylthiazol-2-yl)-2,5-diphenyltetrazolium bromide (MTT) assay (Sigma-Aldrich, St. Louis, MO). Briefly, cells were seeded in 96-well plates (10^4 ^cells per well) and then treated with cytotoxic drugs in the presence and absence of inhibitors. MTT was then added to each well (final concentration, 1 mg/ml) and plates were incubated in a 5% CO_2 _incubator at 37°C for 1 h. Plates were centrifuged at 2,000 rpm for 30 minutes to avoid loss of floating cells. Supernatants were discarded and 150 μl of dimethyl sulfoxide (DMSO), were added to each well. Absorbance was measured at 450 nm.

### Caspase Activity Assays

Caspase activity assays were performed as described previously [43]. Briefly, cells were seeded in black, clear-bottomed 96-well plates (10^4 ^cells per well). At the conclusion of treatment with cytotoxic drugs in the presence and absence of inhibitors, cells were incubated with 50 μl of 3× caspase buffer [150 mM Hepes pH 7.4, 450 mM sodium chloride, 150 mM potassium chloride, 30 mM magnesium chloride, 1.2 mM ethylene glycol-bis(2-aminoethylether)-*N,N,N',N'*-tetraacetic acid (EGTA), 30% sucrose, 10% CHAPS, and 1.5% NP-40], 30 mM dithiothreitol (DTT), 3 mM phenylmethanesulphonylfluoride (PMSF), and 75 μM of the fluorogenic peptide substrates Ac-DEVD-AMC (caspase-3/7) or Ac-VDVAD-AMC (caspase-2) for 2 h at 37°C, followed by incubation at room temperature for 12 h. In these experiments, TRAIL/actinomycin D treatment was used as a control for caspase-3/7 activation, whereas STS was used as a control for caspase-2 activation. Absorbance was then read in a FL_X_800 Microplate Fluorescent Reader (Bio-tek Instruments) at excitation of 360 nm and emission of 460 nm. Fold activity was determined by normalizing to one the absorbance values for untreated cells.

### Immunoblotting

Equal amounts of protein from whole cell lysates were separated by sodium dodecyl sulfate polyacrylamide gel electrophoresis (SDS-PAGE), blotted onto polyvinyl difluoride (PVDF) membranes, and analyzed by immunoblotting as described previously [25].

### Analysis of Mitochondrial Membrane Potential (MMP)

Cells (3 × 10^5 ^per well) were grown in 6-well plates. After treatment with DTX, 7.5 μl of 5,5',6,6'-tetrachloro-1,1',3,3'-tetraethyl-benzimidazoly-carbocyanine iodide (JC-1, stock: 5 mg/ml) were added per ml to each well, followed by incubation at 37°C for 30 minutes. Floating cells were collected and combined with cells harvested by trypsinization. JC-1 retained in 20,000 cells was measured at 530 nm (FL-1, green) and 590 nm (FL-2, red) using a FACScalibur flow cytometer (BD Biosciences). CellQuest Pro software was used for data analysis.

### Flow Cytometric Cell Cycle Analysis

Cells (3 × 10^5 ^per well) were grown in 6-well plates. After treatment with DTX, floating cells were collected and later combined with attached cells harvested by trypsinization. Cells were resuspended in PBS, fixed with 2 ml of ice-cold 70% ethanol, and incubated for 30 minutes at 4°C. Cell pellets were collected by centrifugation (5,000 rpm for 5 minutes) and resuspended in 400 μl of PBS, 50 μl of propidium iodide (PI) solution (0.6 mM) and 50 μl of RNase A (1 mg/ml). After incubation for 30 minutes in the dark at 37°C, cells were analyzed for DNA content using a FACScalibur flow cytometer. Fluorescence from the PI-DNA complex was estimated on a minimum of 50,000 cells per sample and analyzed with CellQuest Pro software.

### Analysis of Lysosomal Membrane Permeabilization

The Acridine Orange (AO) method was used to analyze lysosomal membrane permeabilization (LMP) as described previously [45]. Briefly, cells growing in 6-well culture plates were exposed to AO (5 μg/ml) and counterstained with Hoescht 33342 (1 μg/ml) for 20 minutes at 37°C. Cells were then examined under an Olympus BX50 epifluorescence microscope using a UMPlanPI 60X/0.90W water immersion objective (Olympus). Images were acquired using a digital Spot camera system (Diagnostic Instruments).

### Cathepsin B Activity Assay

Cathepsin B activity was detected using the fluorogenic susbtrate Magic Red MR-(RR)_2 _(Immunochemistry Tech). Briefly, cells growing in 6-well culture plates were exposed for 1 hour to the cathepsin B substrate, rinsed twice with PBS, and counterstained with 1 μg/ml of Hoechst 33342 for nuclear visualization. Cells were directly examined under an Olympus BX50 epifluorescence microscope using a UMPlanPI 60X/0.90W water immersion objective. Images were acquired using a digital Spot camera system.

### Generation of Cell Lines Stably Overexpressing LEDGF/p75

The LEDGF/p75 cDNA was previously cloned into the mammalian expression vector pcDNA3.1+ (Invitrogen) [25]. Both the pcDNA3.1+ empty vector and pcDNA3.1+/LEDGF/p75 vector were transfected into PC3 cells using the Fugene 6 method (Roche). Forty-eight hours post-transfection, cells were trypsinized and seeded into 6-well plates. Selection of stable transfectants was achieved by adding G418 (Fisher Scientific) to the cell cultures. Colonies were expanded and assayed for increased expression of LEDGF/p75 by immunoblotting. Clones overexpressing LEDGF/p75 were selected for further expansion and stored in liquid nitrogen.

### Real-Time PCR

Total RNA was extracted from PC3 cells with TRIzol Reagent (Invitrogen), and single-strandedcDNA was constructed using Superscript III polymerase (Invitrogen) and oligo-dT primers. Real-time polymerase chain reaction (RT-PCR) was performed using the iCycler (Bio-Rad) and SYBR Green PCR master-mix reagents (Abgene). The following primers were used: LEDGF/p75 forward 5'-TCAAGTCAACAGGCAGCAAC-3'; LEDGF/p75 reverse 5'-TAGCTGCAGGTCGTCCTCTT-3'; Cofilin (CFL) forward 5'-AAGTCTTCAACGCCAGAGG A-3'; CFL reverse 5'-GGCCCAGAAGATAAACACCA-3'. Primers were used at a concentration of 4 μM.

## Results

### DTX-induced cell death involves caspase-activation but is not entirely caspase-dependent

To determine whether DTX-induced cytotoxicity in PCa cells is dependent on the activation of caspases, particularly caspase-2, we first examined the activity of caspases-2 and -3 in the hormone-refractory PCa cell lines PC3, DU145, and RWPE-2 after treatment with increasing DTX doses, ranging from 0.01 μM to 5 μM. Since the observed results for these and some subsequent experiments were relatively similar with all the doses and cell lines, and for the sake of brevity, we opted to show only data for PC3 cells treated with relatively low (0.1 μM) and high (3 μM) concentrations. These correspond to plasma DTX concentrations achieved in treated PCa patients during the initial hours post-infusion [12,46].

**Figure 1:**
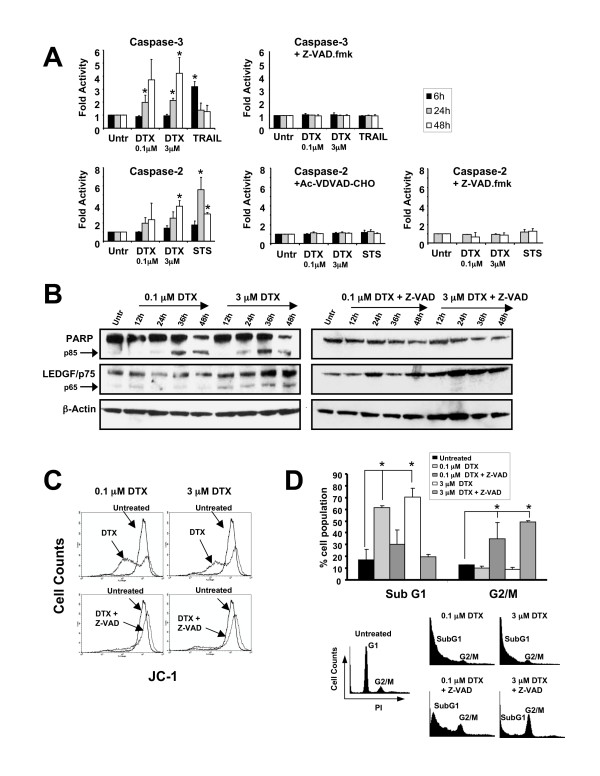
**Docetaxel (DTX) induces caspase-dependent death in PC3 cells**. **A**. Caspase-3/7 and -2 activity assays in PC3 cells treated with DTX, TRAIL, or STS in the presence and absence of the pan caspase inhibitor Z-VAD-FMK and the caspase-2 inhibitor Ac-VDVAD-CHO respectively. Cells were pre-treated 1 h prior to drug treatment. Activity was determined by measuring the cleavage of the fluorogenic substrate Ac-DEVD-AMC for caspase-3/7 and Ac-VDVAD-AMC for caspase-2. Fold activation was determined by normalization of the test sample to untreated controls: **B**. Immunoblotting analysis of PARP and LEDGF/p75 cleavage in DTX-treated PC3 cells in the presence and absence of Z-VAD-FMK: **C**. Flow cytometric analysis of mitochondrial membrane potential (MMP), using the JC-1 method, in PC3 cells treated with DTX in the presence and absence of Z-VAD-FMK for 48 h. A representative of three independent experiments is shown: **D**. Cell cycle analysis of PC3 cells treated with DTX in the presence and absence of Z-VAD-FMK for 48 h. Flow cytometric analysis of cells stained with propidium iodide was used to determine the percentage of cells in SubG1 and G2/M. Representative DNA histograms are shown. Error bars in panels A and D represent the standard deviation of at least three independent experiments done in triplicates (* p < 0.05, t-test).

Both concentrations of DTX induced gradual activation of caspases-2 and 3 during treatment of PC3 cells for up to 48 h (Fig. 1A). STS and TRAIL were used as controls for caspase-2 and caspase-3 activation, respectively (Fig. 1A). Caspase-2 activity was abolished by pre-treatment of cells with the specific inhibitor Ac-VDVAD-CHO, whereas both caspases were completely inhibited with the broad-caspase inhibitor Z-VAD-FMK (Fig. 1A). In light of this, most subsequent experiments were conducted only in the presence of Z-VAD-FMK. We observed that the caspase substrates PARP and LEDGF/p75 [25] were cleaved into their signature apoptotic fragments of p85 and p65, respectively, during DTX-induced PC3 cell death (Fig. 1B). However, no cleavage was observed in the presence of Z-VAD-FMK, consistent with inhibition of caspase activity.

To further highlight the importance of the caspase-dependent pathway in DTX-induced PC3 cell death we analyzed the effect of DTX on mitochondrial membrane potential (MMP). Loss of MMP was induced at both concentrations of DTX after 48 h of exposure to the drug, and was inhibited by Z-VAD-FMK, confirming its dependence on caspase activation (Fig. 1C). We also measured DNA fragmentation by flow cytometric analysis of DNA content. As observed in Fig. 1D, treatment with either 0.1 or 3.0 μM DTX for 48 h led to significant increase in the subG1 cell population. In the presence of Z-VAD-FMK there was a decrease in the subG1 cell population concomitant with increase in the G2/M fraction. Taken together, the data presented in Fig. 1 indicate that both low and high doses of DTX induce caspase activation, caspase substrate cleavage, loss of MMP, and DNA fragmentation. In addition to these events, we also observed that DTX induced reactive oxygen species (ROS) in PC3 cells (data not shown), consistent with a previous report [47].

**Figure 2:**
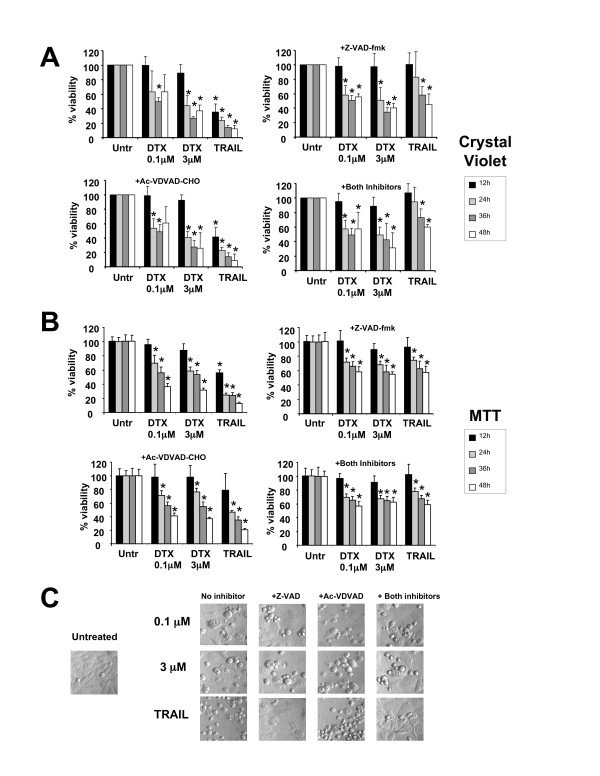
**Inhibition of caspases does not block DTX-induced cytotoxicity in PC3 cells**. **A**. Percentage of surviving PC3 cells treated with DTX in the presence and absence of Z-VAD-FMK, AC-VDVAD-CHO, or both inhibitors. Cells were pre-treated with the inhibitors 1 h before exposure to DTX. Cell viability was determined by crystal violet staining. Absorbance was measured at 570 nm and the values were normalized against those of untreated cells, which were assumed to be 100% viable. Errors bars represent the standard deviation of at least three independent experiments done in triplicate (*p < 0.05 compared to untreated, t-test): **B**. Same as in panel A except that cell viability was measured using the MTT assay. Errors bars represent the standard deviation of two independent experiments done in hexuplicates (*p < 0.05 compared to untreated, t-test): **C**. Morphological analysis of PC3 cells treated as indicated above for 48 h. Cells were visualized on an Olympus IX70 inverted microscope equipped with Hoffmann Modulation Contrast.

To explore the possibility that DTX-induced cell death in PCa cells may also involve a caspase-independent pathway, we first determined whether caspase inhibition would prevent the death of drug-treated PC3 cells. Both low and high concentrations of DTX reduced cell viability as measured by crystal violet staining (Fig. 2A). However, this reduction was only partially abrogated in the presence of the caspase inhibitors Z-VAD-FMK and Ac-VDVAD-CHO, suggesting that a caspase-independent pathway was also activated by DTX. Similar results were observed in DU145 and RWPE-2 cells (data not shown). To confirm that the loss of viability measured in DTX-treated PC3 cells with the crystal violet method was not merely the result of cell rounding and detachment associated with mitotic arrest, we also measured cell viability using a modified MTT method that avoided loss of floating cells, with similar results (Fig. 2B). Consistent with these results, morphological analysis revealed the presence of both rounded and dead cells after treatment with DTX for up to 48 h in the presence of caspase inhibitors (Fig. 2C). However, most cells treated with TRAIL in the presence of caspase inhibitors were still attached and did not display apoptotic morphology (Fig. 2C).

### DTX-induced PC3 cell death involves lysosomal membrane permeabilization

**Figure 3:**
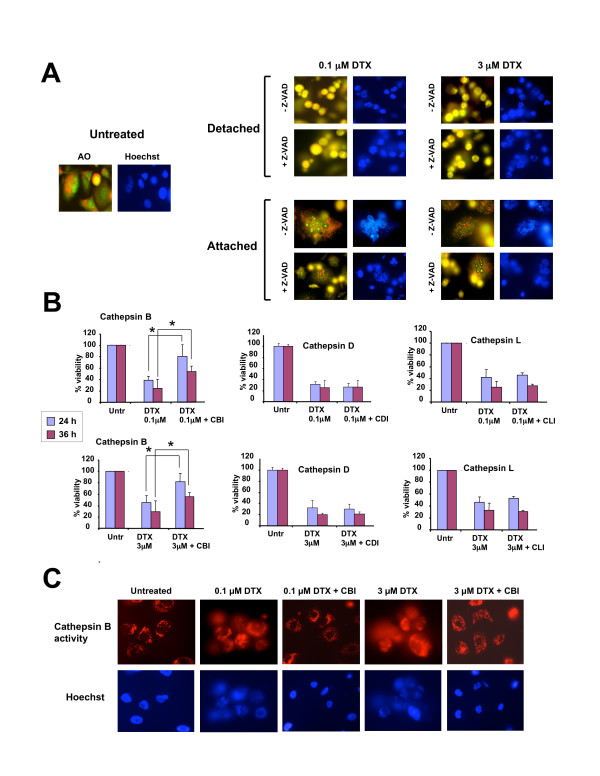
**DTX induces lysosomal membrane permeabilization (LMP) in PC3 cells**. **A**. Determination of lysosomal integrity in PC3 cells treated with DTX for 24 hours in the presence and absence of Z-VAD-FMK. Cells were exposed to acridine orange (AO) for lysosome visualization under fluorescence microscopy. Untreated cells showed localized granular red fluorescence corresponding to staining of intact lysosomes. Cells that detached after treatment with DTX in the presence and absence of Z-VAD-FMK displayed increased yellow fluorescence indicative of LMP. Most cells that remained attached after treatment appeared to have intact lysosomes but exhibited multinucleation. Hoescht staining was used to visualize nuclear morphology: **B**. Cathepsin B inhibitor (CBI) but not inhibitors of cathepsin L (CLI) or cathepsin D (CDI) attenuated cell death induced by 0.1 μM DTX (top set of graphs) and 3 μM DTX (bottom set of graphs), as assessed by crystal violet viability assays. Errors bars represent the standard deviation of at least three independent experiments done in triplicate (*p < 0.05, t-test): **C**. Detection of intracellular cathepsin B activity in PC3 cells using the fluorogenic substrate Magic Red MR-(RR)_2_. Cells treated with DTX for 36 hours showed diffuse cathepsin B activity (red fluorescence), whereas the activity in untreated cells and cells treated with DTX in the presence of CBI was localized mainly to lysosomes.

We evaluated the possibility that DTX might activate the lysosomal cell death pathway concomitant with activation of the caspase-dependent pathway. The acridine orange (AO) method was used to assess lysosomal membrane permeabilization (LMP) in PC3 cells after treatment with 0.1 or 3.0 μM DTX for up to 48 h. Both concentrations of DTX induced extensive AO leakage into the cytosol, producing a diffuse yellow color (Fig. 3A). This was visible mostly in detached cells and was not caspase-dependent since it could not be inhibited by Z-VAD-FMK (Fig. 3A), Ac-VDVAD-CHO (data not shown), or the combination of both inhibitors (data not shown). The attached cells still had intact lysosomes (as visualized by punctuated cytoplasmic red AO staining) but appeared to undergo mitotic catastrophe, as evidenced by the presence of multinucleation (Fig. 3A).

LMP is typically associated with release and activation of cathepsins, particularly cathepsins B, D and L [48,49]. To investigate the involvement of these cathepsins in DTX-induced PC3 cytoxicity, we evaluated whether their inhibition attenuated cell death. For these experiments, PC3 cells were pre-incubated with cathepsin B inhibitor (CA-74Me), cathepsin D inhibitor (pepstatin A), or cathepsin L inhibitor (Cathepsin L inhibitor) prior to exposure to DTX. Inhibition of cathepsins D and L had no effect on PC3 cell viability in the presence of low and high doses of DTX. However, inhibition of cathepsin B significantly increased cell viability (Fig. 3B).

To determine if cathepsin B is released from the lysosomes after treatment of PC3 cells with DTX, a cathepsin B activity assay was performed in which changes in the intracellular localization of the activity of this protease were examined by fluorescence microscopy. Cathepsin B activity was detected using the fluorogenic substrate Magic Red MR-(RR)_2_. This substrate fluoresces once cleaved by cathepsin B. Untreated cells displayed red fluorescence mostly localized to the lysosomes (Fig. 3C). However, cells treated with 0.1 or 3 μM DTX for 36 h displayed diffuse red fluorescence, indicative of cathepsin B activity in the cytosol, most likely resulting from LMP. In the presence of cathepsin B inhibitor there was red fluorescence localized inside the lysosomes in the majority of DTX-treated cells, consistent with attenuation of LMP during DTX-induced cell death. The detection of this red fluorescence also indicated that the inhibitor did not block completely cathepsin B activity.

### Simultaneous inhibition of caspases and cathepsin B antagonizes DTX-induced cell death

**Figure 4:**
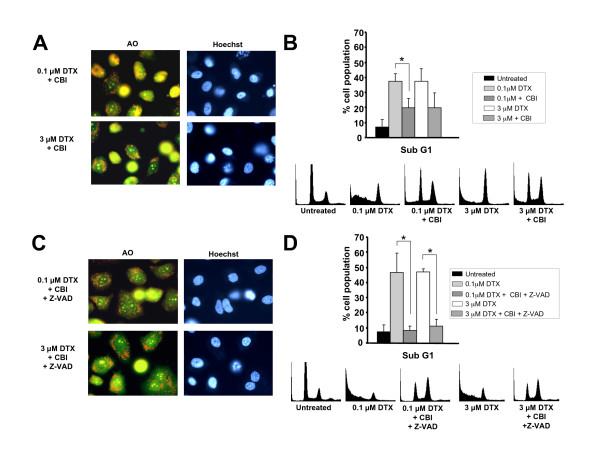
**Simultaneous inhibition of caspases and cathepsin B attenuates DTX-induced cell LMP and DNA fragmentation**. **A**. PC3 cell cultures treated with DTX for 36 hours in the presence of cathepsin B inhibitor and stained with acridine orange (AO) appeared to have less detached yellow cells than cultures treated with DTX in the absence of inhibitor (compare with Fig. 3A). Images were acquired from fields that had both attached and detached cells: **B**. Cell cycle analysis of PC3 cells treated with DTX in the presence and absence of cathepisn B inhibitor for 48 h. Flow cytometric analysis of cells stained with propidium iodide was used to determine the percentage of subG1 cells. Error bars represent the standard deviation of at least three independent experiments (* p < 0.05, t-test). Representative DNA histograms for one of the experiments are shown: **C**. PC3 cell cultures treated with DTX for 36 hours in the presence of both Z-VAD-FMK and cathepsin B inhibitor, and stained with acridine orange, showed very few yellow detached cells, as compared with cultures treated with DTX in the absence of inhibitors (see Fig. 3A): **D**. Cell cycle analysis of PC3 cells treated with DTX in the presence and absence of Z-VAD-FMK and cathepisn B inhibitor for 48 h. Flow cytometric analysis of cells stained with propidium iodide was used to determine the percentage of subG1 cells. Error bars represent the standard deviation of at least three independent experiments (* p < 0.05, t-test). Representative DNA histograms for one of the experiments are shown.

In light of the above results indicating attenuation of DTX-induced cell death by inhibition of cathepsin B, we sought to explore whether blocking cathepsin B activity was sufficient to delay LMP in DTX-treated PC3 cells. Staining of PC3 cells with AO after exposure to DTX and cathepsin B inhibitor for 36 h revealed that while LMP was not as prominent as observed in DTX-treated cells in the absence of inhibitor, there were still yellow cells present, indicating that inhibition of cathepsin B did not confer full protection against DTX-induced cell death (Fig. 4A, compare with Fig. 3A). Since cathepsin B can induce nuclear fragmentation upon release from lysosomes [48], we also examined the effects of cathepsin B inhibition on DNA fragmentation. Inhibition of cathepsin B partially protected against DTX-induced DNA fragmentation, as inferred by the reduction of the subG1 cell population in cultures treated with cathepsin B inhibitor (Fig. 4B). These findings were consistent with the partial protection conferred by cathepsin B inhibitor against DTX-induced cytotoxicity (Fig. 3B).

To determine if both caspases and cathepsin B contribute to LMP, we treated PC3 cells with 0.1 or 3 μM DTX for 36 h in the presence of both Z-VAD-FMK and cathepsin B inhibitor. Interestingly, LMP was markedly decreased in cells treated with both inhibitors, as inferred by the reduction in the number of yellow cells (Fig. 4C). Furthermore, DTX-induced DNA fragmentation was abolished in the presence of both inhibitors (Fig. 4D). We followed the survival of cells treated with DTX in the presence of both Z-VAD-FMK and cathepsin B inhibitor for up to 7 days and noticed that the majority of cells survived as long as both inhibitors were added freshly to cell cultures every 36 h (data not shown). We also observed that inhibition of cathepsin B led to decreased caspase activity, suggesting that LMP and cathepsin activation are upstream events that influence caspase activation (data not shown). Taken together, these results indicated that caspases and cathepsins, particularly cathepsin B, play a concerted role during DTX-induced cytotoxicity.

### Overexpression of LEDGF/p75 attenuates DTX-induced cell death

**Figure 5:**
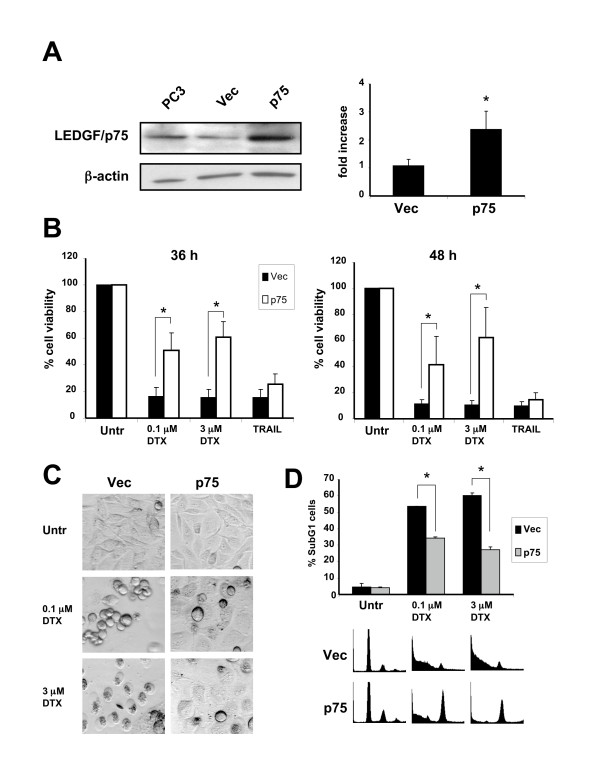
**Overexpression of LEDGF/p75 attenuates DTX-induced cell death**. **A**. LEDGF/p75 expression in parental PC3 cells, pools of PC3 clones stably transfected with empty pcDNA vector (Vec), or pcDNA-ledgfp75 (p75), analyzed by immunoblotting (right panel) and RT-PCR: **B**. Stable overexpression of LEDGF/p75 attenuated DTX-induced cytotoxicity after 36 and 48 h of exposure to low and high concentrations of the drug. Cell survival was determined by crystal violet staining. Data is representative of three independent experiments. Error bars represent the standard deviation of at least three independent experiments done in triplicates (* p < 0.05, t-test): **C**. Morphology of PC3 clones (Vec and p75) treated with DTX for 48 hours. Clones overexpressing LEDGF/p75 showed less dead and detached cells than clones transfected with empty vector: **D**. Cell cycle analysis of PC3 clones (Vec, and p75) treated with DTX for 48 h. Flow cytometric analysis of cells stained with propidium iodide was used to determine the percentage of subG1 cells. Overexpression of LEDGF/p75 significantly attenuated DTX-induced DNA fragmentation. Representative DNA histograms are shown. Error bars represent the standard deviation of at least three independent experiments done in triplicates (* p < 0.05, t-test).

In light of a recent report suggesting that LEDGF/p75 promotes lysosomal stabilization in response to cytotoxic drugs [27], we investigated whether its ectopic expression in PC3 cells would antagonize DTX-induced cytotoxicity and LMP. For these experiments, we generated cell lines stably overexpressing LEDGF/p75 (Fig. 5A). Clones stably expressing LEDGF/p75 displayed increased resistance to 0.1 or 3 μM DTX, when compared to clones transfected with empty pcDNA vector (Fig. 5B). Interestingly, LEDGF/p75 expression did not attenuate TRAIL-induced cell death (Fig. 5B). Consistent with these results, ectopic expression of LEDGF/p75 was also observed to protect RWPE-2 cells against DTX-induced cell death but not against TRAIL or STS-induced cell death (data not shown). Morphological analysis of stable transfectants exposed to DTX for 48 h showed less cell rounding and death in cultures of PC3 cells overexpressing LEDGF/p75 (Fig. 5C). Also, flow cytometric cell cycle analysis revealed a reduction in the percentage of subG1 cells in DTX-treated cultures, consistent with a decrease in the levels of DNA fragmentation (Fig. 5D). However, overexpression of LEDGF/p75 did not abrogate the ability of DTX to induce mitotic arrest, as indicated by the increase in the G2/M fraction in cells treated with 0.1 or 3 μM DTX (Fig. 5D).

**Figure 6:**
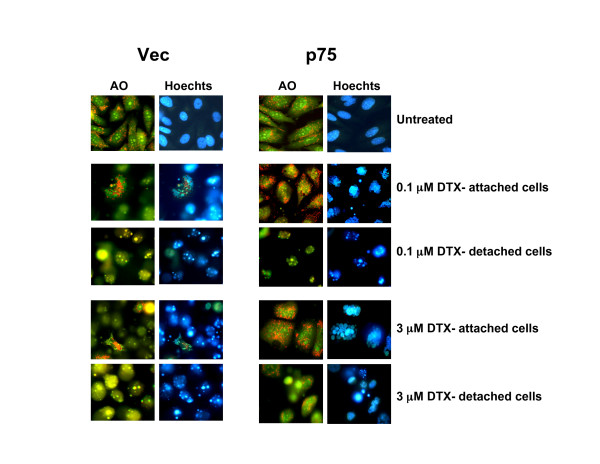
**Overexpression of LEDGF/p75 attenuates DTX-induced LMP**. PC3 cell clones (Vec and p75) were treated with DTX for up to 48 hours and stained with acridine orange (AO) to visualize LMP. Clones stably transfected with empty vector (Vec) showed increased LMP as indicated by minimal or absence of punctuated lysosomal red fluorescence, associated with increased number of detached yellow cells. Clones overexpressing LEDGF/p75 showed less cell detachment and increased lysosomal stability after DTX treatment. These resistant clones displayed extensive multinucleation.

We also investigated whether stable overexpression of LEDGF/p75 stabilized lysosomes in PC3 cells treated with DTX for up to 48 h. Clones overexpressing LEDGF/p75 and treated with DTX appeared to have less detached or apoptotic cells than clones transfected with empty vector (Fig. 6), as assessed by morphological visualization of Acridine Orange-stained cells. Attached cells overexpressing LEDGF/p75 and treated with DTX exhibited stable lysosomes, as evidenced by the presence of cytoplasmic red speckles and absence of yellow-colored cells. However, visual analysis of these attached cells revealed multinucleation, suggesting that ectopic overexpression of LEDGF/p75 attenuates DTX-induced LMP and apoptosis but not mitotic catastrophe.

## Discussion

Several recent studies have established that DTX induces tumor cell death primarily through mitotic catastrophe and apoptosis [11-16]. However, the role of a lysosomal pathway in DTX-induced cell death has not been thoroughly investigated. DTX-induced apoptosis in melanoma and prostate cancer cells has been shown to involve activation of caspases-2 and -3, and changes in MMP [14,16]. Mhaidat et al [14] showed that changes in MMP were almost completely inhibited after caspase-2 knockdown, suggesting that DTX-induced apoptosis is dependent on caspase-2 activation [14]. Hernandez-Vargas et al [15] reported that the coupling of mitotic catastrophe with apoptosis occurs in breast cancer cells only at 0.1 μM (100 nM) concentrations, whereas very low concentrations of 2–4 nM induce mitotic catastrophe followed by a late necrosis. An earlier study by Schimming and colleagues [17] provided evidence that DTX is a potent inducer of mitotic arrest, apoptosis, and necrotic-like tumor cell lysis with pro-inflammatory properties in 15 syngeneic mouse xenografts.

Consistent with these previous findings, our present study demonstrates that DTX induced mitotic catastrophe as well as apoptosis associated with caspase-2 and -3 activation, loss of MMP, and DNA fragmentation in the HRPC cell line PC3. The loss of MMP and the DNA fragmentation were largely caspase-dependent since they were inhibited by Z-VAD-FMK. However, we noticed that although inhibition of caspases-2 and -3 with Ac-VDVAD-CHO and Z-VAD-FMK reduced DTX-induced cytotoxicity, it did not completely block cell death, as assessed by viability assays, morphological visualization, and analysis of LMP. These results indicated that DTX-induced cell death is not entirely dependent on caspase activation since, in addition to mitotic catastrophe and apoptosis, it involves a caspase-independent pathway associated with LMP.

Under our experimental conditions, DTX-induced caspase-independent cell death did not appear to involve necrosis since it was not associated with the generation of the necrotic signature cleavage fragments of PARP and topoisomerase 1 in cells treated with this drug in the presence of Z-VAD-FMK (Fig. 1B and data not shown). In previous reports we described the generation of necrosis-specific fragments of PARP and topoisomerase 1 in cancer cells undergoing primary necrosis, secondary necrosis, and caspase-independent cell death with necrotic morphology [45,50,51]. It is also unlikely that DTX-induced caspase-independent cell death occurs through autophagy, a stress response mechanism triggered by environmental stressors (i.e., starvation, oxidative stress, drugs) which often results in non-apoptotic cell death and involves cathepsin activation and lysosomal dysfunction [22]. We reached this conclusion after immunoblotting analysis did not show a time-dependent upregulation in the expression of the autophagy markers LC3bII and Beclin in DTX-treated PC3 cells (data not shown).

LMP and cathepsin activation are events associated with chemotherapy-induced cell death [48,49]. These events occur during both caspase-dependent and independent cell death, and involve the activation of cathepsins B, D, and L, [48,49]. These proteases contribute to cell death by activating effectors such as mitochondria-associated proteins, caspases, apoptosis-inducing factor (AIF), or by directly cleaving nuclear and cytoplasmic factors [48,49]. Our data suggest that DTX-induced caspase-independent cell death involves LMP associated with cathepsin B activity. Cathepsins D and L do not appear to contribute significantly to this process since their inhibition did not attenuate DTX-induced cell death. These results are consistent with a report implicating LMP and activation of cathepsin B, but not cathepsin D, in caspase-independent cell death induced by the microtubule stabilizing agents paclitaxel, epothilone B, and discodermolide in lung cancer cells [19].

The release of cathepsin B from lysosomes typically follows induction of LMP by agents such as reactive oxygen species, fatty acids, and microtubule toxins [48]. Cathepsin B itself can induce LMP, although the mechanisms are unknown [48]. Once released from the lysosomes, cathepsin B can induce both caspase-dependent and independent cell death, as well as nuclear fragmentation [48]. Interestingly, our data show that inhibition of cathepsin B alone is not sufficient to completely block DTX-induced LMP and DNA fragmentation. However, inhibition of both cathepsin B and caspases led to a dramatic reduction of these events. These findings point to cathepsin B as a key mediator of lysosome-mediated cell death induced by microtubule-stabilizing drugs. They also suggest that DTX-induced cytotoxicity involves the concerted play of caspases and cathepsin B.

Several endogenous inhibitors of lysosome-mediated cell death have been identified [48,49]. Among these, heat-shock protein (Hsp) 70 family members and LEDGF/p75 have recently attracted attention due to their frequent overexpression in tumors, and cytoprotective and chemoresistance properties. Daugaard et al [27] showed that ectopic expression of LEDGF/p75 protected cancer cells against the LMP-inducing agents siramesine and doxorubicin [27]. Our data showed that ectopic overexpression of LEDGF/p75 attenuated DTX-induced cytotoxicity and LMP but had no effect on TRAIL-induced cell death. Overexpression of LEDGF/p75 also attenuated DTX-induced death in RWPE-2 cells but was not effective against TRAIL and STS (data not shown). This suggested that LEDGF/p75 could be a selective inhibitor of cell death, caspase-dependent or independent, associated with LMP. Interestingly, LEDGF/p75 overexpression in PC3 cells failed to prevent DTX-induced multinucleation, suggesting that DTX-induced mitotic catastrophe is not coupled to LMP.

The mechanisms by which LEDGF/p75 promotes lysosomal stability are presently unknown, although the available evidence suggests that this protein may protect cells against oxidative stress by transcriptionally activating stress and antioxidant genes that reduce intracellular ROS [24-26,40,41]. A recent study showed that LEDGF/p75 physically interacts with the oncogenic transcription factor menin and the MLL histone methyltransferase to activate cancer associated genes and promote leukemic transformation [42]. Our group is currently exploring interactions between LEDGF/p75 and various transcription factors to determine if this protein's ability to promote chemoresistance is associated with its capacity to form protein complexes required for transcriptional activation of protective genes.

## Conclusion

This study underscores the ability of DTX to induce concomitantly caspase-dependent and independent death pathways in prostate cancer cells. Our data also point to LEDGF/p75 as a potential contributor to prostate cancer cell resistance to DTX-induced LMP and cell death in vitro. The upregulation of stress and antioxidant genes is emerging as a mechanism associated with tumor chemoresistance. It remains to be investigated if LEDGF/p75 overexpression in human prostate tumors contributes to the upregulation or activity of stress/redox genes associated with cancer progression and chemoresistance. If this turns out to be the case, LEDGF/p75, its protein interacting partners, and its target genes might become attractive candidates for molecular targeting, in combination with DTX and other drugs, in the treatment of HRPC.

## Competing interests

The authors declare that they have no competing interests.

## Authors' contributions

MMV designed and carried out most of the experiments and wrote the initial drafts of the manuscript. FJP carried out the studies on cellular localization of cathepsin B and generated the stable cell lines. FA carried out the studies on mitochondrial membrane permeabilization. JP carried out the western blots of caspase substrate cleavage. ES carried out the RT-PCR experiments on LEDGF/p75 expression. TRD made original observations leading to this work and contributed to the critical revision of the manuscript. LSL conducted imaging analysis of DTX treated cells. AP conducted flow cytometry experiments to determine DTX-induced ROS and helped generate graphs from flow cytometry analysis. NRW supervised cell cycle analyses and contributed to data interpretation. MBL provided valuable reagents and contributed to the critical revision of the manuscript. MDL contributed to the experimental design and critical revision of the manuscript. CAC contributed to the conception and design of the entire study and the final editing of the manuscript.
